# Activation of Cytotoxic Lymphocytes and Presence of Regulatory T Cells in the Trachea of Non-Vaccinated and Vaccinated Chickens as a Recall to an Infectious Laryngotracheitis Virus (ILTV) Challenge

**DOI:** 10.3390/vaccines9080865

**Published:** 2021-08-05

**Authors:** Daniel Maekawa, Sylva M. Riblet, Patrick Whang, David J. Hurley, Maricarmen Garcia

**Affiliations:** 1 Poultry Diagnostic and Research Center, Department of Population Health, College of Veterinary Medicine, University of Georgia, Athens, GA 30602, USA; daniel.maekawa@uga.edu (D.M.); sriblet@uga.edu (S.M.R.); Patrick.Whang@uga.edu (P.W.); 2 Food Animal Health and Management Program, Department of Population Health, College of Veterinary Medicine, University of Georgia, Athens, GA 30602, USA; djhurley@uga.edu

**Keywords:** infectious laryngotracheitis virus (ILTV), chicken embryo origin (CEO), tissue culture origin (TCO), recombinant herpesvirus of Turkey-laryngotracheitis (rHVT-LT), cytotoxic T lymphocytes (CTLs), natural killer (NK) cells, regulatory T cells (Tregs), protection

## Abstract

While the protective efficacy of the infectious laryngotracheitis virus (ILTV) vaccines is well established, little is known about which components of the immune response are associated with effective resistance and vaccine protection. Early studies have pointed to the importance of the T cell-mediated immune responses. This study aimed to evaluate the activation of cytotoxic T lymphocytes (CTLs) and natural killer (NK) cells and to quantify the presence of regulatory T cells (Tregs) in the larynx–trachea of chickens vaccinated with chicken embryo origin (CEO), tissue culture origin (TCO) and recombinant Herpesvirus of Turkey-laryngotracheitis (rHVT-LT) vaccines after challenge. Our results indicated that CEO vaccine protection was characterized by early CTLs and activated CTLs enhanced responses. TCO and rHVT-LT protection were associated with a moderate increase in resting and activated CTLs followed by an enhanced NK cell response. Tregs increase was only detected in the non-vaccinated challenged group, probably to support healing of the severe trachea epithelial damage. Taken together, our results revealed main differences in the cellular immune responses elicited by CEO, TCO, and rHVT-LT vaccination in the upper respiratory tract after challenge, and that activated CTLs rather than NK cells play a main role in vaccine protection.

## 1. Introduction

Infectious laryngotracheitis (ILT) is a highly contagious acute respiratory disease of chickens that results in severe economic losses due to mortality and/or a decrease in egg production. The disease is caused by the avian *Gallid alphaherpesvirus* 1 (GaHV-1) commonly known as infectious laryngotracheitis virus (ILTV). Intervention strategies are directed at control of the disease by implementing strict biosecurity and by vaccination [1]. Currently, two types of live attenuated vaccines (CEO: chicken embryo origin, TCO: tissue culture origin) and recombinant vaccines that use the herpesvirus of turkey (HVT) as vector are mainly used in the United States (US) to control ILT infections. The CEO vaccines, although capable of regaining virulence, induce the best protection against an ILTV challenge. They are the preferred choice for control of severe outbreaks of the disease [2,3]. The TCO vaccine and recombinant HVT-LT (rHVT-LT) vaccines constitute safer alternatives for vaccination than CEO [4,5,6]. Nevertheless, the TCO and rHVT-LT vaccines have been shown to induce a reduced degree of protection than the CEO vaccine in experimental studies [2,3,7] and in the field [8]. Experimentally, CEO vaccines have shown to prevent clinical signs of the disease (dyspnea, conjunctivitis, and lethargy), block challenge virus replication in the trachea [2,3], and prevent virus transmission from vaccinated to naive chickens after challenge [2]. On the other hand, chickens vaccinated with the TCO or the rHVT-LT vaccines, have reduced clinical signs and decreased levels of trachea challenge virus load. Yet, replication of the challenge virus persists in the trachea of chickens vaccinated with either of these vaccines [2,7,9]. Further, challenge virus transmission from rHVT-LT vaccinated chickens to naive chickens has been confirmed experimentally [2,9].

Early studies indicated that T cells rather than humoral responses were responsible for ILT disease resistance [10,11,12,13]. Although there is agreement that cellular immune responses are key to controlling the disease in vaccinated chickens [14], the nature of the components of the protective immune response elicited by vaccination remains unknown. As the larynx and the trachea are principal sites of ILTV replication, it seems prudent to hypothesize that differences in cell-mediated immune responses in these tissues will define the protection elicited by ILT vaccines and set the course for blocking clinical disease. The most prevalent immune cells involved in early viral clearance are cytotoxic lymphocytes, represented by natural killer (NK) cells, and cytotoxic T lymphocytes (CTLs). These cells rapidly bind to and kill virus-infected cells. Both NK cells and CTLs have been previously associated with the clearance of infectious bronchitis virus (IBV) [15], Marek’s disease virus (MDV) [16,17,18], and avian influenza virus (AIV) [19,20]. CTLs carry antigen-specific T cell receptors (TCR) to recognize cognate peptides bound to major histocompatibility complex class-I (MHC-I) antigens [21]. On the other hand, NK cells do not express rearranged antigen receptors but sense their environment via activating and inhibitory receptors that interact with available ligands on the target cell [22]. Despite the differences in how CTLs and NK cells recognize infected cells, in their activated state each cell population will be activated to produce interferon-γ (IFN-γ) and to release cytotoxic granules (including perforin and granzyme) that produce death of infected cells [23]. In chickens, resting NK cells have been described as a population that expresses the surface CD8αα homodimer, but no surface CD3 molecules [24]. During their activation the level of CD8α decreases [25]. In contrast, CTLs express both the CD3 co-receptor and either the CD8αα homodimer or a CD8αβ heterodimer [19,26]. One method for determining the activation state of CTLs or NK cells is by measuring the transient expression of CD107 (LAMP-1) on the surface that occurs during cytotoxic degranulation [27]. Previous studies in chickens have shown that the level of surface expression of CD107 was associated with the level of cytotoxic activity in both CTLs and NK cells in response to avian influenza virus [19] and infectious bronchitis virus [28,29]. Whether CTLs or NK cells are associated with ILTV vaccine protection has not been determined.

In addition to cell-mediated cytotoxicity, cells that trigger and modulate adaptive immune responses are also important in the establishment of long-term immunity against viral pathogens and in maintaining the homeostasis of immune cells in mucosal tissues. T helper (Th) cells are possibly the most important cells in adaptive immunity. They have a required role in many immunologic processes. These include the activation of CTLs and macrophages, maturation and differentiation of B cells, and the recruitment and arming of polymorphonuclear leukocytes (PMNs) [30]. In chickens, Th cells carry the surface marker CD4 and express a T cell-specific surface receptor (TCR) composed of one type of polypeptide heterodimer (α/β) that recognizes viral antigens in association with MHC class II protein [31]. Regulatory T cells (Tregs) are a subset of CD4 T cells that modulate immune homeostasis and establish peripheral tolerance by enforcing negative regulation of T and B cell responses. In the absence of a putative FoxP3 ortholog, chickens Tregs were initially characterized as a CD4^+^ CD25^+^ cell population [32] with the capability to suppress naive T cell proliferation in vitro [33]. Recently, CD4^+^ CD25^+^ and CD4^+^ CD25^−^ Tregs that express membrane-bound TGF-β^+^ have been identified in the lung, cecal tonsils, blood, thymus, and spleen of chickens. This TGF-β^+^ Tregs population significantly increased in the lung early after infection with a virulent Marek’s disease virus (MDV) strain and later in the spleen, during the transformation stage of the disease. Based on this outcome, it was concluded that TGF-β^+^ Tregs escalate the immunosuppression and transformation phases of MDV infection [34]. Besides dampening the efficacy of immune responses, Tregs also play a significant role as a key contributor in resolving tissue inflammation and as mediators of tissue healing in the later phases of multiple viral infections by modulating macrophage activity and survival [35,36]. Whether populations of Tregs are recruited to the trachea after the ILTV challenge of vaccinated and non-vaccinated chickens will provide some initial evidence of the role of these cells in vaccine protection. The objective of this study was to assess the percent of CTLs, activated CTLs, resting and activated NK cells, and Tregs that migrate to the trachea of naïve, CEO, TCO, and rHVT-LT vaccinated chickens after challenge, and how these cell subsets are associated with vaccine protection.

## 2. Materials and Methods

### 2.1. Vaccines and Vaccines Titration

The rHVT-LT vaccine (Innovax-ILT^®^, Merck Animal Health, Madison, NJ, USA) that expresses the glycoproteins D and I of ILTV was reconstituted as recommended by the manufacturers and titrated in a confluent monolayer of secondary chicken embryo fibroblast (CEF) cells. Briefly, CEF cells were seeded in 60 mm cell culture plates at 5 × 10^5^ cells/mL with 5 mL of Ham’s F10 medium (Corning, Corning, NY, USA), 5% fetal bovine serum (FBS) (Atlanta Biologicals Inc, Flowery Branch, GA, USA) and 2% antibiotic-antimycotic (Invitrogen, Waltham, MA, USA). The cell cultures were incubated in a humidified incubator at 37 °C and 5% CO_2_. At 24 h post-incubation, the medium was removed from the plates and replaced with 4 mL of Ham’s F10 medium and 2% FBS. Then, three consecutive 10-fold dilutions of the reconstituted rHVT-LT vaccine were made in Ham’s F10 medium, and 1 mL of each dilution was inoculated to four plate replicates of CEF monolayers. Inoculated CEF plates were incubated at 37 °C and 5% CO_2_. At 5 days post-incubation viral plaques were counted under light microscopy. Virus titers were calculated as plaque-forming unit (PFU) per dose (100 μL) [2]. The CEO vaccine (Laryngo-Vac^®^, Zoetis, Parsippany, NJ, USA) and TCO vaccine (LT-IVAX^®^, Merck Animal Health, Madison, NJ, USA) were also reconstituted as recommended by the manufacturers and titrated in chicken kidney (CK) cells prepared from 3- to 4-week-old specific-pathogen-free (SPF) chickens seeded in 96-well plates as previously described [6]. Briefly, six consecutive 10-fold dilutions (10^−1^ to 10^−6^) of the reconstituted CEO and TCO vaccine were prepared, and five replicates of each dilution were inoculated (100 μL) onto CK cells. Plates were incubated at 39 °C and 5% CO_2_ for 5 days. The 50% tissue culture infective dose (TCID_50_) was determined by the presence of virus-induced cytopathic effect and estimated by the Reed and Muench method [37].

### 2.2. Challenge Virus

The ILTV virulent strain 1874C5 originally isolated from the upper respiratory tract of broilers during outbreaks of the disease and subsequently classified as a member of the genotype VI group [38] was selected as the challenge virus. The challenge strain was propagated in CK cells prepared as previously described [39]. Titers were expressed as tissue culture infective dose (TCID_50_) and calculated by the Reed and Muench method [37].

### 2.3. Experimental Design

Four hundred specific pathogen free (SPF) white leghorn eggs were acquired from a commercial source (Charles River Laboratories Inc., Wilmington, MA) and incubated at 37.5 °C and 55% relative humidity (RH) in a small-scale hatcher (Natureform Inc., Jacksonville, FL, USA) at the Poultry Diagnostic Research Center—University of Georgia. At 19 days of embryonation (doe), eggs were candled, and infertile eggs and dead embryos were removed before transfer to hatcher trays. A total of 225 viable embryos were selected and distributed into five groups of 45 eggs each. One group of 45 embryos were manually vaccinated *in ovo* at 19 doe with a full dose (6500 PFU/100 μL) of the rHVT-LT vaccine. An additional hundred embryos were injected *in ovo* with 0.1% Coomassie blue dye to evaluate the accuracy of the site of injection [40]. During the length of the experiment, chickens were housed in isolation units, with filtered air and negative pressure, and were provided with a standard diet and water ad libitum.

At eight days of age (doa) two groups of 43 chickens each were vaccinated via eye drop with the CEO vaccine at a dose of 10^4.1^ TCID_50_ in 33 μL or with the TCO vaccine at a dose of 10^3.5^ TCID_50_ in 33 μL. At 28 doa, all vaccinated chickens and one group of non-vaccinated (NVx) chickens (n = 43) were challenged (Ch). Groups were identified as rHVT-LT/Ch, TCO/Ch, CEO/Ch, and the NVx/Ch group that served as a positive control. The ILTV virulent strain 1874C5 was administered at a dose of 10^3.8^ TCID_50_ in a total volume of 200 μL split into 50 μL per eye, and 100 μL intra-tracheally. The remaining group of non-vaccinated chickens (n = 43) was mock-inoculated (NCh) with tissue culture media as described above and identified as the NVx/NCh group that served as a negative control. At 1 and 7-days post-challenge (dpch) chickens were humanely euthanized by CO_2_ inhalation and the larynx plus whole trachea were collected from five chickens per group for flow cytometry analysis. At 4 dpch clinical signs scores were obtained from 10 chickens per group. After clinical signs assessment, chickens were humanely euthanized; larynx and whole tracheas were collected from five chickens for flow cytometry analysis and the upper trachea (0.5 cm) was collected for histopathological examination from the other five chickens. 

Five of the 23 remaining chickens per group were pre-selected for collection of tracheal swabs to quantify challenge virus genome load from the same set of chickens at 1, 4, and 7 dpch. Signs of disease were monitored for the 18 residual chickens per group, and survival rates from 1 to 7 dpch were calculated. Humane endpoint was implemented for any chicken with a clinical sign score (total) of 8 to 9. This experiment was conducted under the Animal Use Protocol A2018 06-009-Y2-AO approved by the Animal Care and Use Committee (IACUC) in accordance with regulations of the Office of the Vice President for Research at the University of Georgia.

### 2.4. Clinical Signs

Clinical signs categories such as conjunctivitis, dyspnea, and lethargy were scored on a scale of 0–3 as previously described [2]. Briefly, the scoring system considered: normal: 0, mild: 0.5–1, moderate: 1.5–2, and severe: 2.5–3. Termination by humane endpoint and any mortalities were assigned a score of six. The sum of scores for individual clinical signs categories (total clinical signs) per chicken and the mean clinical sign total score per group at each time point were calculated.

### 2.5. DNA Extraction

Individual tracheal swabs were resuspended in 1 mL of phosphate-buffered saline (PBS) (HyClone Laboratories, Logan, UT, USA) with 2% antibiotic-antimycotic (Gibco, Grand Island, NY, USA) and 2% newborn calf serum (Gibco, Grand Island, NY, USA). Samples were vortexed and stored at −80 °C until further processing. Total DNA was extracted using the MagaZorb^®^ DNA extraction miniprep 96-well kit (Promega, Madison, WI, USA), according to the manufacturer’s recommendations with some modifications [9].

### 2.6. Duplex ILTV Real-Time PCR

Duplex real-time PCR assay that amplifies a fragment of the UL44 viral gene of ILTV and a fragment of the chicken α2 collagen gene was performed as previously reported (Vagnozzi et al., 2012). The relative amount of viral genome load per sample was calculated as the log_10_ 2^−ΔΔCt^ [41].

### 2.7. Histopathology

The upper part of the trachea (0.5 cm) was excised and fixed in 10% buffered formalin. One tracheal ring per chicken was paraffin-embedded and transversally cut into 5 μm sections. Tracheal sections were stained with hematoxylin and eosin (H&E) and the lesion scores were examined under light microscopy. Briefly, each trachea section was individually scored according to the following criteria [42]: Score 0 = normal epithelium, score 1 = normal epithelium with mild to moderate lymphocytic infiltration but no detection of syncytia with intranuclear inclusion bodies, score 2 = normal epithelium with mild to moderate lymphocytic infiltration and few foci of syncytia with intranuclear inclusion bodies, score 3 = affected epithelium with moderate to marked hyperemia and lymphocytic infiltration with numerous syncytia with intranuclear inclusion bodies, score 4 = areas with an absence of epithelium and the occasional presence of syncytia with intranuclear inclusion bodies, score 5 = no residual epithelium remaining and syncytia with intranuclear inclusion bodies rarely found. The mean tracheal lesion scores were calculated per each treatment group.

### 2.8. Single Cell Suspension

The larynx–trachea excised from individual birds were transported from the necropsy area to the laboratory in conical tubes with ice-cold PBS (Gibco, Grand Island, NY, USA) supplemented with 0.5% bovine serum albumin (BSA) (Roche, Basel, Switzerland). Upon arrival to the laboratory, the larynx and trachea were cut into small pieces in the transport media. Subsequently, tissue pieces from single chickens were transferred to conical tubes with enzyme digestion solution composed of DMEM/Nutrient Mixture F-12 Ham (Sigma-Aldrich, St. Louis, MO, USA) supplemented with 1 mg/mL of collagenase D (Roche, Basel, Switzerland), 40 U/mL of DNAse I (Roche, Basel, Switzerland) and 0.1 mg/mL of protease (Sigma-Aldrich, St. Louis, MO, USA). Tissue digestion was performed in an orbital shaker (75 RPM) at 37 °C and 5% CO_2_ for two hours. After digestion, the cell suspension was dispensed through a 40-μm cell strainer (Corning, Corning, NY, USA). A total number of cells and viability was obtained using trypan blue exclusion on a Cellometer Mini (Nexcelcom Bioscience, Lawrence, MA, USA). The final cell count for flow cytometry analysis was adjusted at 6 × 10^6^ cells/mL.

### 2.9. Flow Cytometry

**Figure 1:**
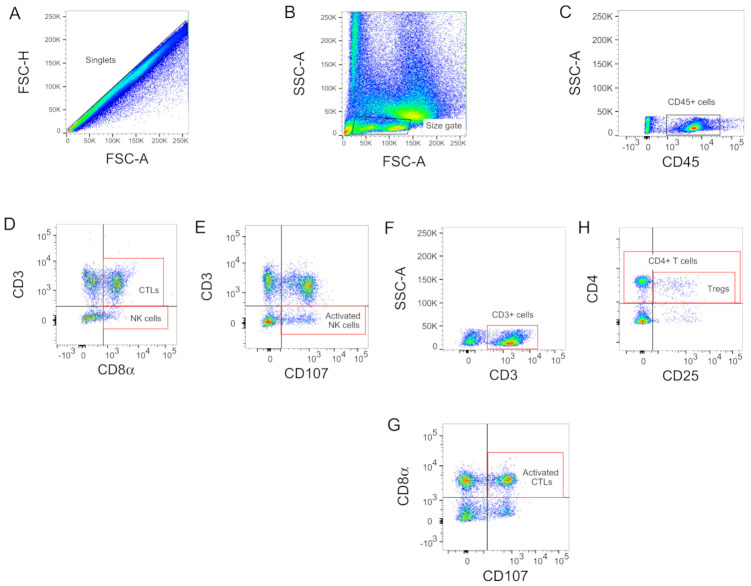
Contour plots of windows and gating strategy used for the identification of immune cells using flow cytometry: (**A**) singlets cells; (**B**) size gate for lymphocytes; (**C**) CD45^+^ cells; (**D**) CTLs (CD3^+^ CD8α^+^) and NK Cells (CD3^−^ CD8α^+^); (**E**) activated NK cells (CD3^−^ CD107^+^); (**F**,**G**) activated CTLs (CD3^+^ CD8α^+^ CD107^+^); (**H**) CD4^+^ T cells and Tregs cells (CD4^+^ CD25^+^).

Enumeration and characterization of avian CTLs (CD3^+^ CD8α^+^), activated CTLs (CD3^+^ CD8α^+^ CD107^+^), NK Cells (CD3^−^ CD8α^+^) and activated NK cells (CD3^−^ CD107^+^) were performed with the following combinations of monoclonal antibodies (mAbs): Mouse anti-chicken CD3-Pacific Blue (CT3; IgG_1_κ), mouse anti-chicken CD8α-FITC (CT8, IgG_1_κ) and mouse anti-chicken CD107-APC (LAMP-1, IgG_1_κ). To identify populations of Tregs (CD4^+^ CD25^+^), mouse anti-chicken CD4-FITC (CT4, IgG_1_κ), and human anti-chicken CD25-Alexa Fluor 647 (AbD13504) mAbs were utilized. To target the leukocyte population and facilitate the identification of CTLs, NK, and Tregs in the larynx–trachea, the mouse anti-chicken CD45-PE (LT40, IgMκ) antibody was included in all staining reactions. All the mAbs except the CD107 (DSHB, University of Iowa, IA, USA) and CD25 (Bio-Rad, Hercules, CA, USA) were obtained from Southern Biotech (Birmingham, AL, USA). All the antibodies dilutions, cell resuspensions (100 μL), and washes (200 μL) were made in PBS supplemented with 0.5% BSA and 1 mM EDTA. Optimal concentration of individual antibodies within the cocktails were pre-determined. Briefly, 100 μL of larynx–trachea cell suspensions (6 × 10^6^ cells/well) and 100 μL of the antibody cocktails were mixed in round-bottom 96-well plates and incubated on ice in a rotator plate shaker (RPMs) for 30 min. After incubation, cells were centrifuged (250 g) at 4 °C for 7 min, washed, resuspended, and stained on ice for 30 min with 100 μL of the aqua LIVE/DEAD fixable stain (Thermo Fisher Scientific, Waltham, MA, USA) for exclusion of dead cells. The stained cells were then centrifuged, washed, resuspended, and fixed with 100 μL of intracellular (IC) fixation buffer (Thermo Fisher Scientific, Waltham, MA, USA). Samples were stored overnight at 4 °C protected from light. In addition, single-stained compensation controls and fluorescence minus one (FMO) controls were included in each flow cytometry run. Flow cytometry evaluations were performed on the BD LSRII flow cytometer (BD Biosciences, Franklin Lakes, NJ, USA). Acquisition and analysis were done using the FACSDiva software version 6 (BD Biosciences) and FlowJo version 10 (TreeStar Inc, Ashland, OR, USA), respectively. Briefly, for the gating strategy (Figure 1), singlets were selected to exclude doublets using the FSC-A versus FSC-H (Figure 1A) and total lymphocytes were size gated based on FSC-A versus SSC-A (Figure 1B). Debris was also excluded from the analysis (FSC-A versus SSC-A). Cells were then subjected to CD45 assessment to establish the leukocyte population (Figure 1C), which was sub-gated to identify CTLs (CD3^+^ CD8α^+^), NK Cells (CD3^−^ CD8α^+^) (Figure 1D), activated NK cells (CD3^−^ CD107^+^) (Figure 1E), activated CTLs (CD3^+^ CD8α^+^ CD107^+^) (Figure 1F,G), CD4^+^ T cells and Tregs cells (CD4^+^ CD25^+^) (Figure 1H). Population frequencies were expressed as a percent of the cell subset of the previous gate (parent gate).

### 2.10. CD107 Assay

The CD107 assay to study NK and CTLs activation was carried out as described previously [19,29]. Briefly, the LAMP-1 hybridoma cell line producing the chicken CD107 (ChCD107) mAb isotype IgG1 was obtained from the Developmental Studies Hybridoma Bank (DSHB, University of Iowa, IA, USA) and was grown according to the DSHB’s recommendations. Once, the ChCD107 mAb was produced, this was purified using spin columns and conjugated with an allophycocyanin (APC) labeling kit (Abcam, Cambridge, MA, USA) following the manufacture recommendations. Single-cell suspensions from larynx–trachea, at a concentration of 6 × 10^6^ cells/mL, were cultured with the ChCD107 mAb for four hours (37 °C and 5% CO_2_) in the presence of the protein transport inhibitor GolgiStop (1 μL/mL) (BD Biosciences, Franklin Lakes, NJ, USA) to prevent degradation of the ChCD107 mAb conjugated fluorochrome [27]. After incubation, cells were washed with PBS supplemented with 0.5% BSA, stained with the mAb mouse anti-chicken CD3 and CD8α, and flow cytometry analysis was performed as previously described.

### 2.11. Statistical Analysis

The assumption of normality for the experimental data was assessed using the Shapiro–Wilk normality test. ILTV viral load, clinical signs, and immune cell percentages were analyzed by one-way ANOVA followed by the post hoc Tukey’s multiple comparison test. Trachea microscopic lesions were compared using the Kruskal–Wallis test and Dunn’s test was performed as a multiple comparison procedure. Correlation analysis between clinical signs scores and immune cells percentages was conducted using the Pearson correlation coefficient. To determine whether the correlation between the two aforementioned variables was significant, the *p*-value was compared to the significant level of 0.05. The Kaplan–Meier method was used for building survival curves, and differences were analyzed using the log-rank sum test. All statistical analyses were performed using the GraphPad Prism software 8 (GraphPad Software Inc, La Jolla, CA, USA). Statistical differences were defined at a significance level of *p* < 0.05. All reported *p*-values were from two-sided comparisons.

## 3. Results

### 3.1. CEO Vaccine Showed the Best Protection against an ILTV Challenge

**Figure 2:**
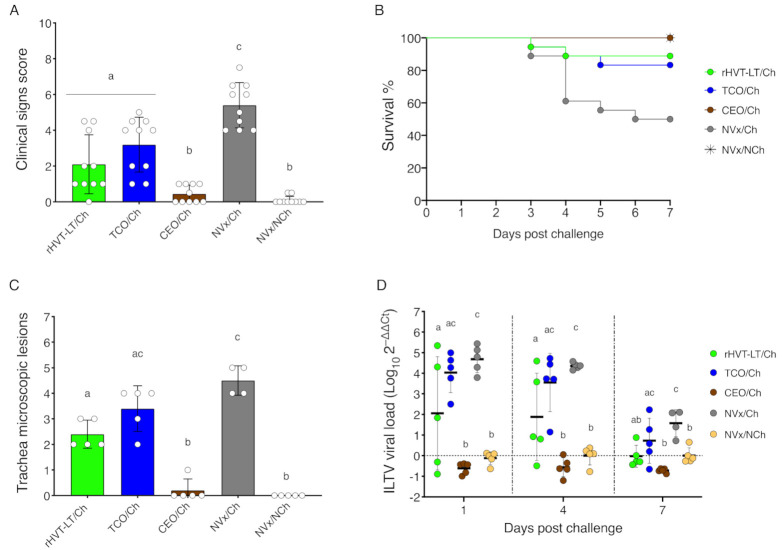
Vaccines induce protection against an ILTV challenge: (**A**) clinical signs score at 4 dpch; (**B**) survival percentage from 1 to 7 dpch; (**C**) histopathology lesions score in the trachea at 4 dpch; (**D**) ILTV viral load in the trachea at 1, 4 and 7 dpch. Bar graphs and small horizontal lines indicate the mean (±SD). Each dot represents an individual chicken. Different letters indicate significant differences (*p* < 0.05).

ILTV vaccine protection was assessed by the ability of the vaccinated chickens to reduce or block clinical signs, mortality, challenge virus replication, and microscopic lesions in the trachea after challenge (Figure 2). At 4 dpch, clinical signs were significantly reduced in all the vaccinated groups compared to the NVx/Ch group (*p* < 0.05). However, among the vaccines, the CEO/Ch group showed the lowest clinical signs (*p* < 0.05), with similar results to the NVx/NCh group (Figure 2A). The absence of clinical signs in the CEO/Ch group was further reflected with a survival rate of 100%, whereas in the TCO/Ch and rHVT-LT/Ch group, the survival rates were significantly reduced to 83.3% and 88.9% (*p* < 0.05), respectively. As expected, the lowest survival rate was observed for the NVx/Ch group (50.0%) (Figure 2B). At 4 dpch, tracheal microscopic lesions were significantly lower in the CEO/Ch group compared to the NVx/Ch, rHVT-LT/Ch, and TCO/Ch groups (*p* < 0.05) (Figure 2C). In addition, the CEO vaccinated chickens were the only group to block the cytolytic replication of the challenge virus and epithelial integrity of the trachea epithelium was maintained in this group. Presence of viral genomes in the trachea was evaluated at 1, 4, and 7 dpch by PCR. Among the vaccinated groups, the CEO/Ch group showed the lowest presence of challenge virus genomes in the trachea, which was comparable to the NVx/NCh. The rHVT-LT vaccinated group of chickens, compared to NVx/Ch group (*p* < 0.05), was able to reduce challenge virus genome load in the trachea, whereas the TCO/Ch group wasn’t (Figure 2D). Overall, the CEO vaccine is considered to induce complete protection against an ILTV challenge, as the protection parameters aforementioned were similar to the NVx/NCh group, while the TCO and rHVT-LT vaccines were categorized to induce partial protection by their ability to confer better results than the NVx/Ch group but not equivalent to the NVx/NCh group.

### 3.2. Early Increase of CTLs and Activated CTLs in Vaccinated Challenged Groups of Chickens

**Figure 3:**
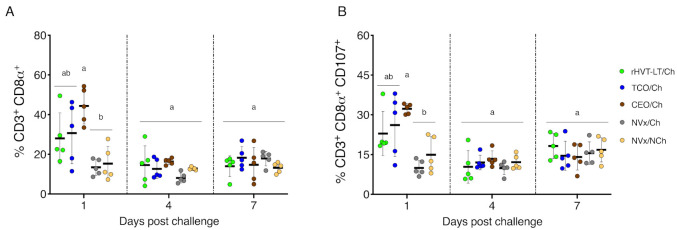
CTLs and activated CTLs in the larynx–trachea at 1, 4 and 7 dpch: (**A**) CTLs (CD3^+^ CD8α^+^); (**B**) activated CTLs (CD3^+^ CD8α^+^ CD107^+^). Small horizontal lines indicate the mean (±SD). Each dot represents an individual chicken (n = 5 for each group). Different letters indicate significant differences (*p* < 0.05).

At 1 dpch, the percentage of CTLs (CD3^+^ CD8α^+^) (Figure 3A) and activated CTLs (CD8α^+^ CD107^+^) (Figure 3B) in the larynx–trachea were significantly increased in the CEO/Ch group compared to NVx/Ch and NVx/NCh group (*p* < 0.05). In addition, the percentage of CTLs and activated CTLs in the rHVT-LT/Ch and TCO/Ch groups, although increased, were not statistically different from the NVx/Ch and NVx/NCh group. At 4 and 7 dpch no significant differences in the percentages of CTLs and activated CTLs were observed among the vaccinated challenged groups and the controls (Figure 3A,B).

### 3.3. Late Increase of NK and Activated NK Cells in the Non-Vaccinated Challenged Group of Chickens

**Figure 4:**
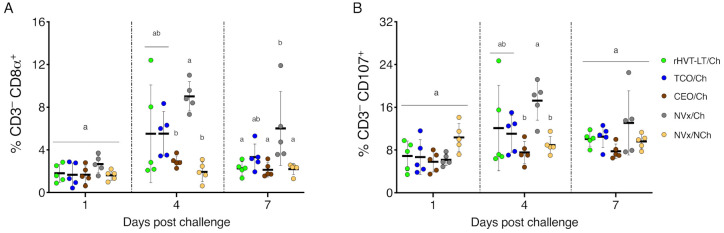
NK and activated NK cells in the larynx–trachea at 1, 4 and 7 dpch: (**A**) NK cells (CD3^−^ CD8α^+^); (**B**) activated NK cells (CD3^−^ CD107^+^). Small horizontal lines indicate the mean (±SD). Each dot represents an individual chicken (n = 5 for each group). Different letters indicate significant differences (*p* < 0.05).

No significant differences in the percentage of NK (CD3^−^ CD8α^+^) (Figure 4A) and activated NK (CD3^−^ CD107^+^) (Figure 4B) cells in the larynx–trachea were detected among the vaccinated challenged groups and the NVx/Ch and NVx/NCh controls by 1 dpch. At 4 dpch, the percentage of NK cells and activated NK cells were significantly higher in the NVx/Ch group than in the CEO/Ch and NVx/NCh groups of chickens (*p* < 0.05); whereas, the percentages of NK and activated NK cells of rHVT-LT/Ch and TCO/Ch vaccinated groups, although higher, were statistically similar to the CEO/Ch and NVx/NCh groups of chickens (Figure 4A,B). At 7 dpch, the percentage of NK cells in the NVx/Ch group was still higher than the percentages detected in the rHVT-LT/Ch, CEO/Ch, and NVx/NCh groups of chickens (*p* < 0.05), while the percentage of NK cells in the TCO/Ch group of chickens was not significantly different than the NVx/Ch group (Figure 4A). At 7 dpch the percentage of activated NK cells was similar among vaccinated challenged groups and the NVx/NCh and NVx/Ch groups (Figure 4B).

### 3.4. Increase of Tregs Populations Only Observed in the Non-Vaccinated Challenged Group of Chickens

**Figure 5:**
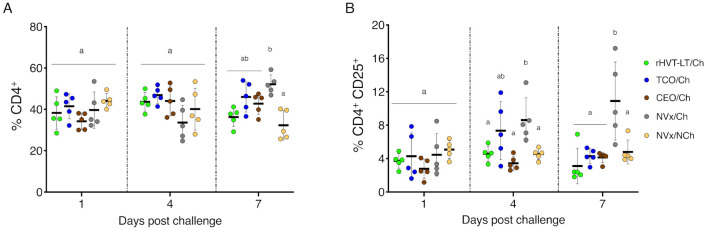
CD4^+^ T cells and Tregs in the larynx–trachea at 1, 4 and 7 dpch. (**A**) CD4^+^ T cells; (**B**) Tregs (CD4^+^ CD25^+^). Small horizontal lines indicate the mean (±SD). Each dot represents an individual chicken (n = 5 for each group). Different letters indicate significant differences (*p* < 0.05).

At 1 dpch, no significant differences in the percentages of CD4^+^ T cells (Figure 5A) and Tregs (CD4^+^ CD25^+^) (Figure 5B) in the larynx–trachea were observed among the vaccinated challenged, the NVx/NCh, and NVx/Ch groups of chickens. At 4 dpch, although no changes in the percentage of CD4^+^ T cells were detected among vaccinated challenged, NVx/Ch, and NVx/NCh groups, the percentages of Tregs were significantly increased in the NVx/Ch group (*p* < 0.05), and were not different to the TCO/Ch group (Figure 5B). At 7 dpch, the percentage of CD4^+^ T cells was significantly higher in the NVx/Ch group compared to the NVx/NCh group (*p* < 0.05); however, no significant differences in CD4^+^ T cells were observed among vaccinated groups (Figure 5A). Similarly, at 7 dpch, the percentage of Tregs was increased in the NVx/Ch group and showing appreciable differences with all vaccinated challenged groups and the NVx/NCh group of chickens (*p* < 0.05) (Figure 5B).

### 3.5. Reduction of Clinical Signs in Vaccinated Groups of Chickens Correlate with Increased Percent of CTLs

**Figure 6:**
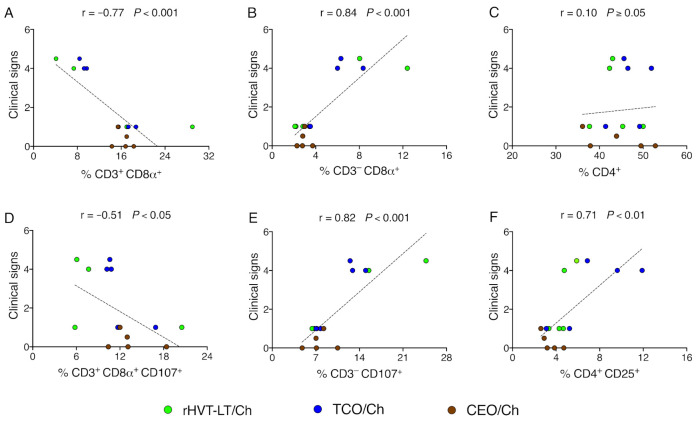
Correlation between clinical signs scores from vaccinated challenged chickens and percentage of immune cells (%) at 4 dpch: (**A**) CTLs; (**B**) NK cells; (**C**) CD4^+^ T cells; (**D**) activated CTLs; (**E**) activated NK cells; (**F**) Tregs. Each dot represents an individual chicken; n = 15 for each correlation, where 5 chickens were included for each vaccinated challenged group; r value represents the Pearson correlation coefficient. An overlapping data point is sometimes observed. Statistical differences are defined at a significance level of *p* < 0.05.

Clinical signs scores collected from vaccinated challenged groups of chickens were correlated with the percentage of CTLs, activated CTLs, NK cells, activated NK cells, CD4^+^ T cells, and Tregs in the larynx–trachea evaluated at 4 dpch. A strong positive correlation was observed between increased clinical signs and the percentages of NK cells (r = 0.84) (Figure 6B), activated NK cells (r = 0.82) (Figure 6E) and Tregs (r = 0.71) (Figure 6F). On the other hand, the reduced presence of clinical signs was strongly and moderated negatively correlated with the percentages of CTLs (r = −0.77) (Figure 6A) and activated CTLs (r = −0.51) (Figure 6D), respectively. Neither a positive or negative correlation was present between clinical signs and CD4^+^ T cells (r = 0.10) (Figure 6C).

## 4. Discussion

This study aimed to assess the percent of CTLs, activated CTLs, resting and activated NK cells, and Tregs found in the larynx–trachea after challenge of CEO, TCO, and rHVT-LT vaccinated chickens and how these cell subsets are associated with vaccine protection. In agreement with previous studies, in this work the CEO vaccinated group of chickens showed optimal protection post-challenge as demonstrated by prevention of clinical signs and mortalities, and complete reduction of the challenge virus replication and lesions in the trachea. For rHVT-LT and TCO vaccinated chickens, although clinical signs and challenge virus replication in the trachea were reduced as compared to the non-vaccinated control, at four days post-challenge virus replication persisted in the trachea inducing moderate trachea lesions. As expected from a primary infection with a virulent ILTV strain, the non-vaccinated group of chickens presented a reduced survival rate (50%), severe clinical signs, extensive virus replication, and utter tracheal epithelial damage.

T cell population changes in the trachea were detected at one day post-challenge and corresponded to increases in the percentage of CTLs and activated CTLs in vaccinated chickens. In contrast to vaccinated groups, no increase of CTLs and activated CTLs were detected in the larynx–trachea of the non-vaccinated group of chickens. The percent of CTLs and activated CTLs in the trachea of CEO vaccinated chickens were higher than those detected in the trachea of TCO and rHVT vaccinated groups. Since collection of single-cell suspensions from the larynx–trachea, and the collection of tracheal swabs from the same chicken was not compatible, performing correlation analysis between CTLs percentages and challenge virus genome load was not possible in this study. Instead, comparisons of individual clinical signs scores from vaccinated challenged groups of chickens to the percentage of CTLs (r = −0.77, *p* < 0.001) and activated CTLs (r = −0.51, *p* < 0.05) at day four post-challenge (peak of clinical signs) indicated a significant negative correlation (Figure 6A,D). Although this correlation analysis was performed following the plateau of the percent of CTLs among groups of vaccinated chickens, it still revealed that increased magnitude of CTLs and activated CTLs in the larynx–trachea was correlated with a reduction in clinical signs in vaccinated chickens. 

The expansion of CTLs observed at day one post-challenge in the vaccinated groups do not exclude an even earlier onset of T cells that may arise from resident memory T cells programmed to promptly respond to antigen [43]. In the case of CEO vaccinated chickens, complete ablation of challenge virus replication at day one post-challenge coincided with the expansion of CTLs and activated CTLs in the trachea. On the other hand, although there was an expansion of CTLs and activated CTLs in the trachea of rHVT-LT and TCO vaccinated group of chickens, this expansion was not sufficient to completely block challenge virus replication. Evidence suggests that inherent differences between parental strains and mode of attenuation of the CEO and TCO vaccines might influence the level of effective memory CTLs responses elicited upon challenge. CEO vaccines are characterized by a faster onset of replication and transmission than the TCO vaccine [6]. It has been shown that upon ocular administration, the CEO vaccines induce lytic replication in the conjunctiva epithelium, and viral genomes are detected in the trachea in the absence of viral lytic replication. In contrast, after ocular administration the TCO vaccine did not produce lesions characteristic of viral lytic replication in the conjunctiva, and viral genomes were not detected in the trachea [44]. Therefore, based on their aggressive replication, it can be speculated that the CEO vaccines are more effective in inducing antigen stimulation and local tissue damage which might result in a stronger memory CTLs response. It has been demonstrated that tissue-resident memory CD8^+^ T cells of the mouse female reproductive mucosae secrete cytokines that trigger rapid adaptive and innate immune responses capable to achieve near sterilizing immunity upon antigen re-stimulation [45]. In this study, although the level of replication of the CEO and TCO vaccines after ocular administration was not evaluated, the lower impact on challenge virus replication of the TCO vaccinated group suggests that the TCO vaccine replicated poorly in the conjunctiva and trachea epithelium. Consequently, upon antigen re-stimulation, inferior CTLs recall responses were achieved. Unlike the CEO vaccine, it is believed that the inability of the rHVT-LT vector to effectively replicate in the upper and lower respiratory tract limits antigen stimulation in these sites [46]. The HVT vector replicates systemically and establishes latency in T cells [47,48,49]. Results from this study indicate that upon challenge the moderate increase of CTLs in the trachea of rHVT-LT vaccinated chickens contributed to the decrease of challenge virus replication. Is not exactly known how the activated CTLs response was induced in the trachea of rHVT-LT vaccinated chickens. We speculate that *in ovo* vaccination with the HVT vector expressing ILTV antigens establishes latency in T lymphocytes, some of these T lymphocytes re-circulate to the trachea and other mucosal tissues and stimulate an immune response.

Other changes detected in the trachea cell subsets were for resting and activated NK cells at four days post-challenge in non-vaccinated, TCO, and rHVT-LT vaccinated groups of chickens. The percent of resting and activated NK cells in the trachea of non-vaccinated chickens was higher than the percent detected in the trachea of rHVT-LT and TCO vaccinated chickens after challenge. Previous studies have shown increased frequencies of activated NK cells in the lungs of chickens infected with low pathogenic avian influenza (LPAI) virus, where the increase coincided with the decline of viral load by six days post-infection [19]. Therefore, it was not surprising to observe an increase of activated NK cells in the larynx–trachea of non-vaccinated chickens at four days post-challenge followed by a decline in challenge virus by day seven post-challenge. On the other hand, the moderate increases of resting and activated NK cells in the trachea of TCO and rHVT-LT vaccinated chickens were unexpected at four days post-challenge. Comparison of individual clinical signs scores from vaccinated groups of chickens to the percentages of resting (r = 0.84, *p*  < 0.001) and activated NK cells (r = 0.82, *p*  < 0.001) at day four post-challenge indicated a significant positive correlation (Figure 6B,E). The positive association between the increase in clinical signs and the percent of NK cells indicated that activation of NK cells in TCO, and rHVT-LT vaccinated chickens were not responses elicited by vaccination, but rather an innate driven response designed to suppress ongoing challenge virus replication. It has been documented that upon replication, Alphaherpesviruses use multiple strategies to avoid the CTLs responses [50]. For example, MDV infections downregulate the MHC class I molecule BF2 which restricts antigen-specific CTLs responses but promotes NK cell activation [51]. Whether ILTV infection downregulates CTLs recognition and favors NK cells activation needs to be further examined.

No increases in CD4^+^ T cells were detected among vaccinated groups after the challenge. Only, on day seven post-challenge, the percent of CD4^+^ T cells was increased in the larynx–trachea of the non-vaccinated chickens. Further, no association was revealed between clinical signs of the disease and the magnitude of CD4^+^ T cells found in the trachea of vaccinated chickens (Figure 6C). It has been recently shown that vaccination with MDV CVI988 induced CD8α^+^ but not CD4^+^ T cells to the pool of CD3^+^ memory T cells [52]. Finally, a significant increase in the percent of Tregs (CD4^+^ CD25^+^) cells was detected in the trachea of non-vaccinated group of chickens from day four to seven post-challenge. Although the magnitude of Tregs was not directly compared to microscopic trachea lesion scores, probably this T cell subset was recalled primarily as a response to dampen the damage associated with inflammatory responses and to support healing of the trachea epithelial.

## 5. Conclusions

This study is the first to present a comprehensive analysis of CD8+ cell subsets activation and changes in Tregs in the trachea of ILTV vaccinated (CEO, TCO, rHVT-LT) and non-vaccinated chickens after challenge. Protection elicited by the CEO vaccine was principally associated with the early increases of CTLs and activated CTLs. In contrast, in partially protected rHVT-LT and TCO vaccinated chickens activation of NK cells was necessary to lessened virus replication. Finally, for the non-vaccinated chickens, increased recruitment of Tregs after the challenge was probably a response to dampen tissue damage and mediate tissue repair.

## Data Availability

The data presented in this study are available on request from the corresponding author.
